# A novel approach to surface electromyography: an exploratory study of electrode-pair selection based on signal characteristics

**DOI:** 10.1186/1743-0003-9-24

**Published:** 2012-04-26

**Authors:** Cynthia Kendell, Edward D Lemaire, Yves Losier, Adam Wilson, Adrian Chan, Bernie Hudgins

**Affiliations:** 1 The Ottawa Hospital Rehabilitation Centre, 505 Smyth Road, Ottawa, ON, K1H8M2, Canada; 2 Institute of Biomedical Engineering, UNB, 25 Dineen Drive, Fredericton, NB, E3B5A3, Canada; 3 Department of Systems and Computer Engineering, Carleton University, 1125 Colonel By Drive, Ottawa, Ontario, K1S 5B6, Canada

**Keywords:** Surface electromyography, Electrode array, Time-domain parameters, Frequency-domain parameters

## Abstract

A 3 × 4 electrode array was placed over each of seven muscles and surface electromyography (sEMG) data were collected during isometric contractions. For each array, nine bipolar electrode pairs were formed off-line and sEMG parameters were calculated and evaluated based on repeatability across trials and comparison to an anatomically placed electrode pair. The use of time-domain parameters for the selection of an electrode pair from within a grid-like array may improve upon existing electrode placement methodologies.

## Background

In physical rehabilitation, surface electromyography (sEMG) is a useful clinical tool for making evidence-based decisions on muscle function, muscle activation timing, and interventions that will affect muscle contraction. New approaches to sEMG data collection could improve the quality of captured sEMG signals, especially for clinicians who only use sEMG periodically. Since electrode placement is important for obtaining an sEMG signal with maximum information from the muscle, improved methods for achieving appropriate electrode placement will enhance outcomes based on these signals.

An sEMG signal can be described in terms of amplitude and frequency. Signal amplitude is typically used as a measure of relative force production and increases with the number, size, and firing rate of active motor units. The frequency content of sEMG is dependent on the characteristics of active muscle fibers, such as size, conduction velocity, and firing rate. When collecting sEMG, the depth of the active motor units, thickness of the subcutaneous tissues, and proximity to the innervation zone and tendons affect measures of amplitude and frequency. As such, electrode placement plays a crucial role in sEMG signal quality.

For decades, studies have investigated optimal electrode placement strategies [1-9]. Typically, the recommended location for electrode placement is the muscle belly, halfway between the motor end plate and the tendinous insertion and away from the lateral borders of the muscle [10]. As a result, several guidelines for optimal electrode placement have been published in which anatomical landmarks are used to locate the muscle belly [4,5,9]. Locating the muscle belly can be challenging given that muscle location, in relation to anatomical landmarks, may vary between subjects and the ability to locate these landmarks may vary between practitioners, depending on their skill and experience level. Using this method for electrode placement is also problematic when individuals require monitoring of muscle function over time and consistent electrode placement is required.

Other studies have based electrode placement recommendations on the innervation zone (IZ) location [1-3,6-8,11]. Identified using a linear electrode array, the IZ was found to be a poor location for electrode placement because the signal over this area is not representative of EMG activity in the rest of the muscle [1-3,6,8,11]. While locating the IZ would indicate where to avoid collecting sEMG, identifying the IZ is in itself a time consuming and complex process, particularly when multi-muscle sEMG is required. As such, this approach would not be practical in a clinical setting where time is limited.

Typically, EMG data collection guidelines aim to increase signal amplitude while minimizing noise, thus increasing the signal-to-noise ratio [10]. The goal of finding a high amplitude signal was also reported by Zipp [9] who wrote that electrode placement should meet three criteria: 1) repeatability, 2) consideration of individual body dimensions, and 3) a high signal yield (i.e., high amplitude). Higher amplitude signals could be used to identify the area of the muscle with the most activity. In addition, maximum mean and median power spectral frequencies (MNF and MDF, respectively) are associated with areas near the IZ [6]. In the IZ, action potentials are incomplete or non-propagating, therefore shorter in duration and lower in amplitude than propagating action potentials [12]. The action potentials also tend to move in opposite directions away from the IZ, resulting in signals that are similar in shape but opposite in phase [13,14]. Due to differential amplification, signal amplitude over the IZ will be substantially reduced [6].

The work reported in this paper is a part of an ongoing research effort that is exploring the use of an electrode array to simplify electrode placement. Instead of attempting to place a single electrode pair optimally, which is a time-consuming process, an electrode array is placed on top of the muscle of interest and an automated method is used to select an optimal electrode pair based on sEMG signal characteristics. The electrode array could be integrated in a wearable sleeve to enable quick placement of the array. The objective of this study is to evaluate commonly used sEMG parameters for assessing the quality of electrode placement, as an initial step towards developing a method to automatically select an optimal electrode pair from an electrode array. Parameters are evaluated based on their repeatability across trials, demonstrating parameter consistency. The appropriateness of this approach will also be examined by comparing the proposed methods of electrode selection to the traditional method of electrode placement using only anatomical landmarks.

## Methods

### Subjects

A convenience sample of eight individuals, six males and two females, was recruited from the University of New Brunswick. The average participant characteristics were: age = 27.6 years (sd = 7.3), weight = 81.0 kg (sd = 23.5), height = 174.6 cm (sd = 10.0). The Research Ethics Board of the University of New Brunswick approved the experimental procedure used for this research and each subject provided informed consent prior to data collection.

### Data collection

**Figure 1:**
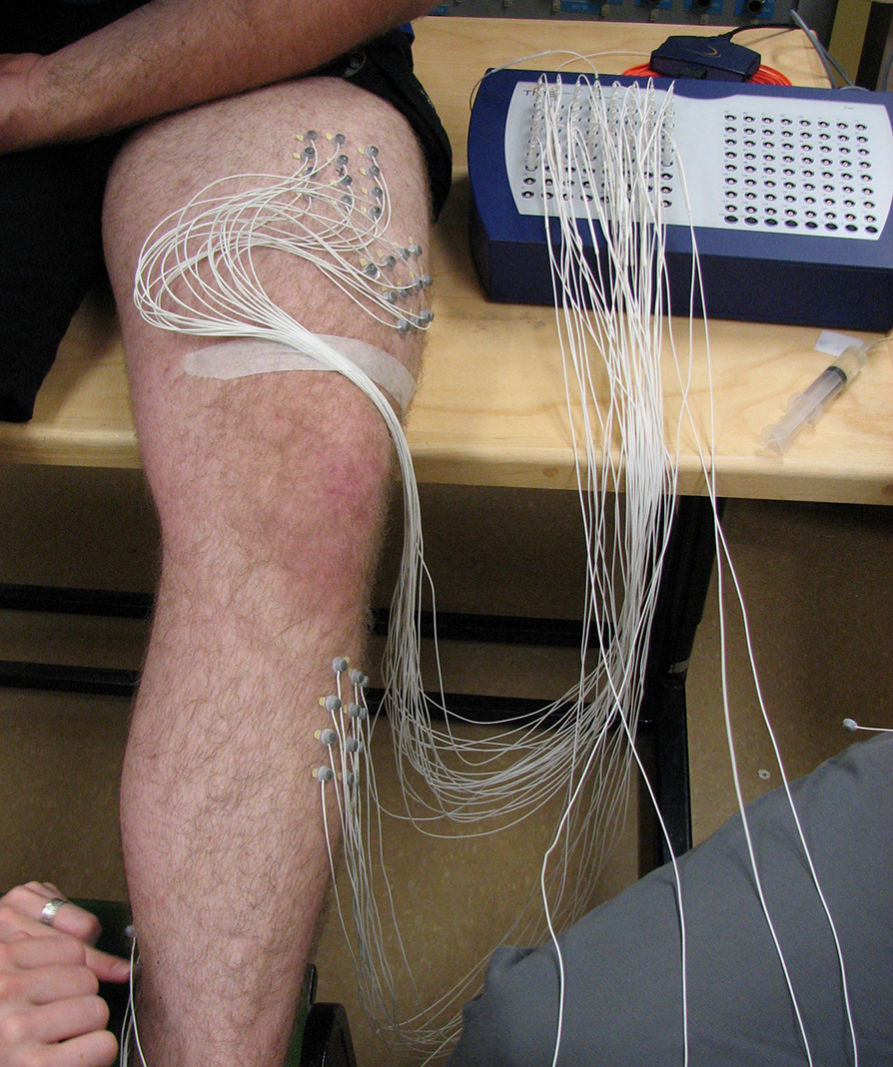
The application of electrode arrays.

Data were collected in the Biosignals Laboratory at the Institute of Biomedical Engineering, University of New Brunswick. A REFA multi-channel amplifier system (TMS International) and proprietary software (PortiLab2) was used for sEMG data acquisition. The REFA system specifications are listed in Table 1. Two-millimeter diameter recessed sintered Ag-AgCl disc electrodes with driven shielded cable were filled with electrode gel, attached to the skin using double-sided tape, and used to simultaneously collect 84 channels of monopolar EMG data (Figure 1).The REFA system amplifier gains were set at 20x and the system incorporates anti-alias filters with 6.786 kHz cutoff frequencies, samples the inputs at 131 kHz and then passes the sampled signals through 5th order SINC filters with 26.7 kHz cutoffs. The collected data were then post-processed with the PortiLab software to be low-pass filtered at 500 Hz with a first-order digital IIR filter, high-pass filtered at 10 Hz with a first-order digital IIR filter and down-sampled to 2048 Hz.

**Table 1:** REFA specifications (TMS International)

**Parameter**	**Specification**
Input Impedance	> 10^12^
CMRR	> 100 dB
Gain	20 V/V
Noise	1.0μVrms
Anti-Alias Filter	1st order RC filter, cutoff frequency = 6.786KHz
Low Pass Digital Filter	5th order SINC filter, cutoff frequency = 0.2035 * sample frequency
Analog to Digital Converter	22 bit resolution sigma delta

**Figure 2:**
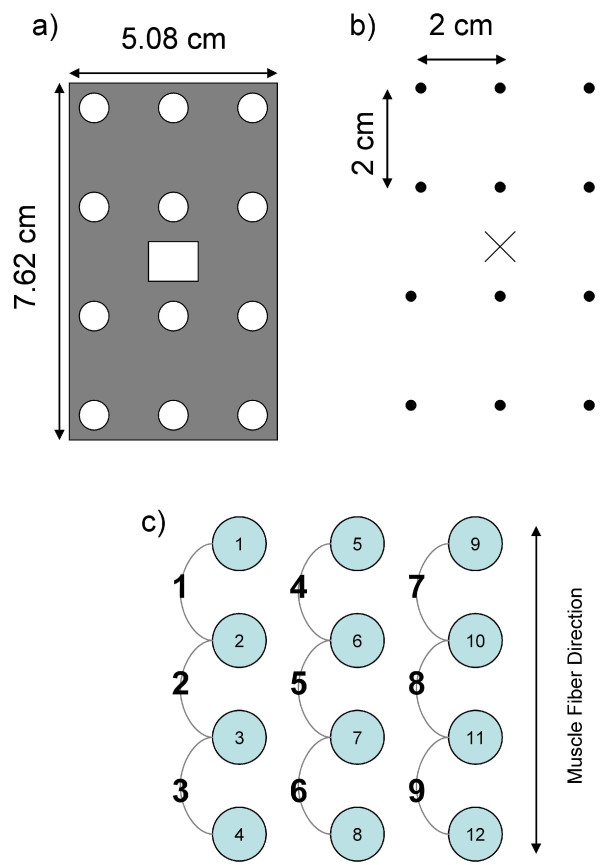
**Array placement and electrode pair formation.a)** The grid used to mark electrode locations; **(b)** Marks made on the skin using the grid. The "x" denotes the guideline-recommended electrode location, while the dots are used to mark where electrodes are placed; **(c)** The formation of 9 bipolar electrode pairs from 12 monopolar electrodes.

For each subject, the skin was cleaned with alcohol pads prior to electrode placement. Due to the small electrode size, hair was not an issue so the skin was not shaved. Using the SENIAM electrode placement guidelines [5] the recommended electrode position was located for seven muscles and marked with an "x" on the skin. The muscles included: tibialis anterior (TA), gastrocnemius medialis (GM), gastrocnemius lateralis (GL), vastus lateralis (VL), rectus femoris (RF), biceps femoris (BF), and semitendinosus (ST). As shown in Figure 2a, a grid was centered on the "x", and ink dots were made on the skin (Figure 2b). An electrode was placed on each dot to form a 3 × 4 electrode array over each muscle (Figure 2c). The array was positioned such that potential electrode pairs were oriented parallel to the direction of the muscle fibres.

Four tasks were chosen to target specific muscles (Table 2). For each trial, five seconds of data were collected during a maximal isometric contraction. To ensure that all electrodes were functioning properly throughout data collection, a resting trial was recorded before and after data collection. Subjects were permitted a rest period between trials to minimize any fatigue effect.

**Table 2:** Description of tasks performed by subjects and the muscles targeted

**Task**	**Description**	**Muscle(s)**
Resisted Plantarflexion	Subjects gripped the edge of a heavily weighted table (approximately 200 Kg). Subjects were asked to generate a maximal contraction by plantarflexing against the weight of the table (i.e., attempting to lift the table using only plantarflexion).	GM
		GL
Resisted Dorsiflexion	Subjects stood with both feet on the ground. A research assistant used both hands to push downward against the dorsal surface of the foot. Subjects were asked to generate a maximal contraction by dorsiflexing against the resistance provided by the research assistant.	TA
Resisted Knee Extension	Subjects stood on one leg with the contralateral knee flexed to 90 °. A research assistant sat behind the subject and held the foot to maintain knee flexion. The subject was then asked to generate a maximal contraction by attempting to extend the knee against the resistance provided by the research assistant.	RF
		VL
Resisted Knee Flexion	Subjects stood on one leg with the contralateral knee flexed to 90 °. A research assistant sat behind the subject and placed both hands over the heel of the flexed limb. The subject was asked to generate a maximal contraction by attempting to flex the knee against the resistance provided by the research assistant.	BF
		ST

### Data analysis

Each array contained 12 electrodes. Since monopolar electrodes were used, nine electrode pairs were defined (Figure 2c). The difference in voltage was calculated to yield the bipolar EMG signal for each pair.

A total of 280 trials (8 subjects × 7 muscles/subject × 5 trials/muscle) were analyzed using Matlab (Mathworks, Natick MA). For each trial, 5 s of data were used to calculate twelve parameters. Six of the parameters were time domain parameters: root mean square (RMS), mean absolute value (MAV), maximum amplitude (MAX), slope sign changes (SSC) [15], zero crossing (ZC) [15], and waveform length (WL) [15]. Time domain parameters were extracted from the raw EMG data, except for MAX. The MAX value corresponded to the maximum amplitude of the EMG envelope, obtained by low pass filtering the fully rectified EMG signal with a 6^th^ order Butterworth filter with a cutoff frequency of 5 Hz.

The remaining six parameters were spectral parameters: mean frequency (MNF) [16], median frequency (MDF) [17], signal-to-motion artifact ratio (SMR), maximum-to-minimum drop in power density ratio (DPR), signal-to-noise ratio (SNR), and the power spectrum deformation (Ω); the latter four spectral parameters were used previously to quantify EMG signal quality [18] and are described in further detail in Appendix A. The EMG power spectral density (PSD) was computed via Welch’s averaged modified periodogram, with 1024 sample analysis windows, overlapping by 50%.

For all trials and all parameters, each pair within an array was ranked based on the value for each parameter, with 1 being assigned to the electrode pair with the best value for that parameter and 9 being assigned to the pair with the worst value. MNF and MDF were found to be higher near the IZ [6], so a rank of 1 was assigned to the electrode pair with the lowest value. Similarly, a rank of 1 was assigned for the electrode pair with the lowest power spectrum deformation (Ω). For all other parameters, the electrode pairs with the maximum values were ranked as 1.

#### Repeatability

A parameter was considered repeatable, for a particular muscle and a particular subject, if an electrode pair had a consistent ranking of 1. For each parameter, for all subjects and all muscles, the number of times that the parameter met the repeatability criteria was counted. The repeatability-4 score is the number of electrode pairs that had a ranking of 1 for at least 4 of 5 trials. The repeatability-5 score is the number of electrode pairs that had a ranking of 1 for all 5 trials. A percentage value was calculated based on how many times the condition of repeatability was met out of the 56 possible cases (8 subjects × 7 muscles).

#### Inter-parameter agreement

For each subject and muscle, all parameters that were identified as repeatable, using the repeatability-4 criteria, were compared against all the other parameters for each trial. If another parameter had the same electrode pair, with a ranking of 1 for at least 3 of the 5 trials, there was inter-parameter agreement between these parameters for that subject and muscle. Inter-parameter agreement was computed between all parameters, resulting in a 12 × 12 inter-parameter agreement matrix. The value on the diagonal of the matrix corresponds to a parameter compared against itself, and therefore should equal the repeatability value of that parameter.

#### Agreement

If the electrode pair corresponding to the guideline-recommended electrode position (hereafter referred to as the guideline-recommend electrode pair) was the same as the electrode pair with a ranking of 1, electrode placement was considered to be in agreement. For each subject and muscle (8 subjects × 7 muscles = 56 cases), the case was considered in agreement if the guideline-recommended electrode pair and the top ranked pair were the same for 4 out of 5 trials.

## Results

### Repeatability

Table 3 shows the repeatability of each parameter. The parameter with the highest repeatability-4 was RMS amplitude (82.14%) and the least repeatable parameter was SMR (7.14%). For the time domain parameters, RMS (82.14%), MAV (78.57%), and WL (80.36%) had the highest repeatability-4 values. For the spectral parameters, MNF (75.00%) and MDF (75.00%) had highest repeatability-4 values, which were slightly lower than the highest time domain parameters.

**Table 3:** Repeatability for each EMG parameter

**Parameter**	Repeatability-4^*^		**Repeatability-5**^**†**^	
	**of 56 cases**	**%**	**of 56 cases**	**%**
**Root mean square (RMS)**	46	82.14	40	71.43
**Mean absolute value (MAV)**	44	78.57	40	71.43
**Maximum amplitude (MAX)**	39	69.64	29	51.79
**Slope sign changes (SSC)**	31	55.36	21	37.50
**Zero crossings (ZC)**	29	51.79	18	32.14
**Waveform length (WL)**	45	80.36	38	67.86
**Mean Frequency (MNF)**	42	75.00	24	42.86
**Median Frequency (MDF)**	42	75.00	21	37.5
**Signal-to-motion artifact ratio (SMR)**	4	7.14	1	1.79
**Maximum-to-minimum drop in power density ratio (DPR)**	26	46.43	17	30.36
**Signal-to-noise ratio (SNR)**	36	64.29	21	37.50
**Power spectrum deformation (Ω)**	30	53.57	20	35.71

^*^ number of cases where the top ranked (i.e., ranked 1) electrode pair was the same for at least 4/5 trials

^**†**^ number of cases where the top ranked electrode pair was the same for all 5 trials.

Repeatability-5 has a stricter requirement than repeatability-4, and Table 3 shows an expected decrease in all values from repeatability-4 to repeatability-5. The highest valued repeatability-4 time domain parameters (RMS, MAV, WL) did not decrease as much as the highest valued repeatability-4 frequency domain parameters (MNF, MDF).

### Inter-parameter agreement

Table 4 shows the inter-parameter agreement matrix. There was perfect inter-parameter agreement between RMS and MAV and strong inter-parameter agreement between MAX and both RMS and MAV. Although the WL repeatability value was high, inter-parameter agreement was not as strong as RMS, MAV, and MAX. MNF and MDF also had high repeatability values and appear to have inter-parameter agreement with each other, although not a strong agreement.

**Table 4:** Inter-parameter agreement matrix

**Parameter**	**RMS**	**MAV**	**MAX**	**SSC**	**ZC**	**WL**	**MNF**	**MDF**	**SMR**	**DPR**	**SNR**	**Ω**
**Root mean square (RMS)**	46	46	44	0	0	28	13	7	7	21	25	9
**Mean absolute value (MAV)**	44	44	42	0	0	27	13	8	7	22	25	9
**Maximum amplitude (MAX)**	38	39	39	0	0	24	12	7	4	18	23	7
**Slope sign changes (SSC)**	0	0	0	31	20	4	0	1	2	0	0	0
**Zero crossings (ZC)**	0	0	0	19	29	7	0	0	3	0	0	5
**Waveform length (WL)**	30	27	27	6	6	45	5	2	8	12	13	8
**Mean Frequency (MNF)**	13	14	13	0	0	5	42	30	5	20	17	5
**Median Frequency (MDF)**	6	7	7	2	1	1	34	42	4	13	11	1
**Signal-to-motion artifact ratio (SMR)**	2	3	3	1	1	2	0	0	4	2	2	1
**Maximum-to-minimum drop in power density ratio (DPR)**	12	14	14	0	0	5	12	9	4	26	25	8
**Signal-to-noise ratio (SNR)**	20	21	19	0	0	11	14	10	6	30	36	12
**Power spectrum deformation (Ω)**	8	7	7	2	5	8	1	0	5	7	10	30

Each row considers cases that are considered repeatable based on the repeatability-4 criteria for a given parameter. The values indicate how many of these cases had a ranking of 1, for the same electrode pair, in at least 3 of the 5 trials for another parameter (column).

### Agreement

The percentage agreement between each parameter and the guideline-recommended electrode pair is presented in Table 5. Agreement for all parameters was low, with a maximum of 16.07% for WL. When all parameters were considered, the average agreement was only 8.48%.

**Table 5:** Agreement by parameter

**Parameter**	**Agreement**	
	**of 56 cases**	**%**
**Root mean square (RMS)**	8	14.29
**Mean absolute value (MAV)**	7	12.5
**Maximum amplitude (MAX)**	7	12.5
**Slope sign changes (SSC)**	7	12.5
**Zero crossings (ZC)**	7	12.5
**Waveform length (WL)**	9	16.07
**Mean Frequency (MNF)**	2	3.57
**Median Frequency (MDF)**	1	1.79
**Signal-to-motion artifact ratio (SMR)**	0	0
**Maximum-to-minimum drop in power density ratio (DPR)**	3	5.36
**Signal-to-noise ratio (SNR)**	4	7.14
**Power spectrum deformation (Ω)**	3	5.36

The condition for agreement was met when the guideline-recommended electrode pair was the same as the electrode pair that had a ranking of 1.

## Discussion

Selecting the best sEMG channel based on amplitude parameters is straightforward in principle; the channel/pair with the highest amplitude are due to electrodes being placed over the most active muscle area. Since amplitude is lower over the IZ, choosing the best pair based on maximum amplitude parameters would reduce the possibility of choosing an electrode pair over the IZ. Also, by increasing amplitude, the signal-to-noise ratio is effectively increased.

For sEMG data collection, repeatability of the measured signal given common measurement conditions is essential. Based on repeatability between five trials, across all subjects and lower extremity muscles, six parameters had repeatability-4 values greater than 75%. From the highest repeatability value to lowest, these six parameters were RMS, WL, MAV, MNF, MDF, and MAX. Only RMS and MAV had a repeatability-5 value greater than 70%, suggesting that these parameters are more robust.

Good inter-parameter agreement was found between RMS and MAV, which is expected since they are both proportional to the average EMG amplitude (RMS corresponds to square root of the average square EMG magnitude and MAV corresponds to the average absolute magnitude). There was also good inter-parameter agreement between these parameters and the MAX parameter; however, the agreement was weaker than RMS-MAV. Since the MAX parameter corresponds to a single value over the EMG signal, rather than an average, its value is susceptible to higher variability and would be expected to be less reliable. The lower repeatability value associated with MAX reinforces this statement. Also, the WL parameter has only a modest inter-parameter agreement with RMS, MAV, and MAX, despite these parameters all having good repeatability values. WL is associated with the EMG signal derivative, computed as the sum of the absolute difference between adjacent samples. This single parameter provides a measure of waveform amplitude, frequency, and duration [15]. Its sensitivity to frequency content may explain why WL only had a modest inter-parameter agreement with the amplitude measures.

The spectral parameters associated with EMG signal quality (i.e., SMR, DPR, SNR, and Ω) exhibited low repeatability. The EMG data used in this study were from isometric contractions in a laboratory setup; therefore, EMG signal quality was expected to be quite high in all cases. Indeed, the mean ± standard deviation of these parameters across all cases were: SMR 26.25 ± 12.46 dB, DPR 42.25 ± 7.19 dB, SNR 30.99 ± 5.57 dB, and Ω 0.73 ± 0.17. While these parameters do not appear to be beneficial for identifying an optimal electrode pair, they could be useful for excluding EMG channels with a low signal quality.

There is a correlation between amplitude and frequency content as a function of electrode position, with EMG from the IZ corresponding to a minimum in amplitude and maximum in MNF and MDF [6]; however, our results showed a low inter-parameter agreement between amplitude parameters (RMS, MAV, and MAX) and the MNF and MDF spectral parameters. These spectral parameters may have been more sensitive to electrode position along the muscle fiber axis than the orthogonal direction. Repeatability values of the spectral parameters decreased much more than the time domain parameters from repeatability-4 to repeatability-5. This suggests that time domain parameters would provide a more consistent method for electrode selection. Larger differences were observed with amplitude parameters than spectral parameters as a function of distance from the IZ [6], which also suggests that amplitude parameters can be expected to be more repeatable.

It is important to note that muscle geometry is an important factor in EMG [19-21]. For example, muscle fibers may also have inhomogeneities due to various fiber orientations [21]. In the context of this work, the influence of the IZ on surface EMG may not be as prevalent in pinnate muscles (e.g., GM, GL, VL, and RF), where muscle fibers insert at an oblique angle to the tendon, compared to fusiform muscles (e.g., TA, BF, and ST); however, IZ effects are still observable in surface EMG measurements [11].

Agreement was examined to determine whether the electrode pair placed over the guideline-recommended location was the same as the top ranked pair for any of the twelve parameters. For all parameters, agreement was very low. This suggests that an array approach has a better chance of successfully locating an electrode pair based on maximal output as compared to the standard anatomical approach.

The electrode array approach could be considered for cases where the clinician has difficulty locating anatomical landmarks, such as for people with thick adipose tissue. The array could also be beneficial for test-retest situations where consistent electrode placement over time is difficult to reproduce. Variations in the EMG due to electrode positioning can be a confounding factor in analysis. In [22], an electrode array was also used for measurement of EMG from the lower extremities to assess this variability in the context of gait analysis. While the objective of this work is different, the results are consistent in that certain electrode pairs exhibited less variability than others. In addition, appreciable differences in robustness were observed between electrode pairs within the array, despite the array being placed near those adopted in clinical practice. An automatic electrode array approach would also provide results that are independent of a person’s experience with electrode placement; such as in home care or isolated healthcare environments.

## Conclusion

This study supports a new approach for sEMG data collection. Typically, an electrode pair is located on the muscle and then data are collected from that site. The proposed method involves collecting EMG data from multiple sites on the same muscle and using signal characteristics to choose the electrode pair at the best location (i.e., location that most consistently provides the strongest signal).

An electrode array approach offers a more accurate and repeatable method of locating the best site for sEMG data collection. The results of this study demonstrated that RMS appears to be the best parameter to provide a quantitative measure for electrode selection. Other parameters, such as WL, MNF, and MDF, had also high repeatability but low inter-parameter agreement with RMS, which suggests that these parameters can provide additional information that could be integrated with RMS. A multi-parameter approach is anticipated to increase the reliability and repeatability of electrode selection.

## Appendix

The following four spectral indices, briefly described below, were developed by Sinderby *et al*. and a complete description can be found in their paper [18].

## Signal-to-motion artifact ratio (SMR)

SMR assumes that motion artifact manifests itself at frequencies below 20 Hz and that the uncontaminated EMG power spectrum is fairly linear between 0 and 20 Hz. The SMR was computed as a ratio of the sum of all power densities for frequencies below 600 Hz and the sum of all power densities that exceed a straight line between the axis origin and the highest mean power density value, with a frequency above 35 Hz. The mean power density, which represents a smoothed version of the PSD, was obtained by averaging PSD values over 13 consecutive points; in this work, the PSD of the EMG data (f_s_ = 2048 Hz) was estimated via Welch’s averaged modified periodogram, with 1024 sample analysis windows, overlapping by 50%, so 13 consecutive points represents a span of 26 Hz (13 Hz above and below the frequency of interest).

## Maximum-to-minimum drop in power density ratio (DPR)

DPR is the ratio of the highest mean power density value and lowest mean power density value, with a frequency between 35 and 600 Hz. The mean power density was obtained by averaging PSD values over 13 consecutive points.

## Signal-to-noise ratio (SNR)

SNR is a ratio of the signal power and noise power. The signal power was estimated as the sum of all power densities for all frequencies below 1000 Hz. The noise power was estimated by determining the average power density between 500 and 1000 Hz and multiplying it by the entire frequency range (i.e. 1000 Hz). Note that this parameter was noted to produce falsely high values for noise in the low frequency range (e.g. motion artifact) [18].

## Power spectrum deformation (Ω)

The Ω ratio is sensitive to changes in spectral symmetry and provides a indication of spectral deformation. It is computed as:

(1) $$\Omega =\frac{\sqrt{M_{2}/M_{0}}}{M_{1}/M_{0}}$$

M_n_ is the n^th^ spectral moment defined as:

(2) $$M_{n}=\sum_{i=0}^{i_{\max}}P_{i}f_{i}^{n}$$

where P_i_ is the power spectral density value at frequency f_i_.

## Competing interests

The authors declare that they have no competing interests.

## Authors’ contributions

CK was involved in study design, data collection, data processing/analysis, and in drafting the manuscript. EL conceptualized the study, developed the protocol, was involved in data processing and analysis, and assisted in drafting the manuscript. YL contributed to the study design, data collection, and analysis. AW contributed to the study design and data collection. AC was involved in signal processing and data analysis as well as writing the manuscript. BH contributed to study conceptualization, and interpretation. All authors critically revised the manuscript for important intellectual content and have read an approved the final draft.
